# An On-Ice Measurement Approach to Analyse the Biomechanics of Ice Hockey Skating

**DOI:** 10.1371/journal.pone.0127324

**Published:** 2015-05-14

**Authors:** Erica Buckeridge, Marc C. LeVangie, Bernd Stetter, Sandro R. Nigg, Benno M. Nigg

**Affiliations:** Human Performance Laboratory, Faculty of Kinesiology, University of Calgary, Calgary, Alberta, Canada; West Virginia University, UNITED STATES

## Abstract

Skating is a fundamental movement in ice hockey; however little research has been conducted within the field of hockey skating biomechanics due to the difficulties of on-ice data collection. In this study a novel on-ice measurement approach was tested for reliability, and subsequently implemented to investigate the forward skating technique, as well as technique differences across skill levels. Nine high caliber (High) and nine low caliber (Low) hockey players performed 30m forward skating trials. A 3D accelerometer was mounted to the right skate for the purpose of stride detection, with the 2nd and 6th strides defined as acceleration and steady-state, respectively. The activity of five lower extremity muscles was recorded using surface electromyography. Biaxial electro-goniometers were used to quantify hip and knee angles, and in-skate plantar force was measured using instrumented insoles. Reliability was assessed with the coefficient of multiple correlation, which demonstrated moderate (r>0.65) to excellent (r>0.95) scores across selected measured variables. Greater plantar-flexor muscle activity and hip extension were evident during acceleration strides, while steady state strides exhibited greater knee extensor activity and hip abduction range of motion (*p*<0.05). High caliber exhibited greater hip range of motion and forefoot force application (*p*<0.05). The successful implementation of this on-ice mobile measurement approach offers potential for athlete monitoring, biofeedback and training advice.

## Introduction

Skating is the typical movement in ice hockey and is the foundation upon which other important ice hockey skills are built. Excellent skating ability is considered one of the main characteristics of a highly skilled ice hockey player [1]. In order to improve skating performance a biomechanical understanding of the variables that have the largest contribution to skating performance is essential. It is assumed that identifying the biomechanical differences between elite and recreational level ice hockey skating can be used to determine the variables that contribute to high level skating performance. However, research into ice hockey biomechanics, particularly relating to movement and actions of the lower limbs, is scarce with little information regarding the biomechanical variables that influence performance available [2,3].

The lack of knowledge of skating biomechanics may be due to the dynamic characteristics of ice hockey and its unique on-ice conditions, as this can make data collection protocols extremely challenging to implement [4]. As such, previous studies have investigated hockey skating biomechanics on smaller scales [2] using skating treadmills [4] or on synthetic ice [3]. However, such laboratory settings impose restrictions on the movements that can be executed, with the artificial environment of skating treadmills or synthetic ice surfaces calling into question the validity of the data. For example, a comparison of over ground and treadmill running showed considerable kinematic differences, with subjects’ foot strike patterns systematically differing between the two conditions [5]. Similar investigations have also been performed in ice hockey, for comparisons of skating on synthetic ice and traditional ice. Despite good agreement being found overall, synthetic ice was found to promote greater knee extension during forward skating [3]. In a comparison of treadmill and on-ice skating, many variables including stride rate, stride length, heart rate and VO_2_ were significantly different [6]. These results endorse the need to improve external validity through on-ice data collection. Furthermore, movements that can be performed on a treadmill are restricted to linear, cyclic motions. However, a game of ice hockey consists of accelerations, changes in direction, sudden stopping and crossovers, and these movements cannot be readily captured within a restricted laboratory volume. This further underlies the importance of measuring biomechanical data on the ice, where there is potential to measure such movements.

A limited number of studies have provided insight into caliber differences with respect to the biomechanics of ice hockey skating. For example, Upjohn et al. [4] investigated differences in three-dimensional lower limb kinematics between high and low caliber hockey players on a skating treadmill. They found substantial differences between groups, with greater stride lengths, segmental excursions and increased joint range of motion in high caliber players. Muscle activity of the lower limbs during hockey skating across different treadmill velocities has also been examined, where an increase in velocity was shown to result in increased activation of the hip flexors, knee extensors, and ankle plantar flexors [7]. Kinematic data has also been recorded during on-ice hockey skating using portable high-speed cameras installed on a railing system [2]. It was found that as hockey players reached the third stride, skating technique shifted towards a gliding motion, as characterised by larger knee flexion amplitudes. This supports 3D kinematic data of male elite speed skaters, where de Koning et al. [8] reported substantial differences between push-off kinematics during initial strides of a sprint, compared to the latter phase, where the strides transition from a ‘running-like’ technique to a ‘gliding’ motion.

Identifying these changes in kinematics and muscle activity provides preliminary information regarding the biomechanics of hockey skating. However, no study to date has recorded a combination of kinematic, kinetic, insole pressure and muscle activity data during forward skating to obtain a comprehensive understanding of biomechanical skating technique on-ice. Furthermore, biomechanical differences between elite and recreational hockey players are not well understood, particularly the ways in which skill level modulates biomechanical variables that have a large contribution to skating performance. This information could further be used to develop biomechanical feedback systems and mobile coaching tools that have the potential to train important skating skills. Therefore, the purposes of this study were to (1) quantify the reliability of a wireless and portable measurement approach that enables comprehensive biomechanical data collection on ice, (2) quantify the biomechanical differences between the acceleration (ACC) and steady-state (SS) phases of forward skating, and (3) examine the effect of player caliber (High vs. Low) on performance and biomechanics of maximum effort forward skating.

## Materials and Methods

### Subjects and Protocol

**Fig 1:**
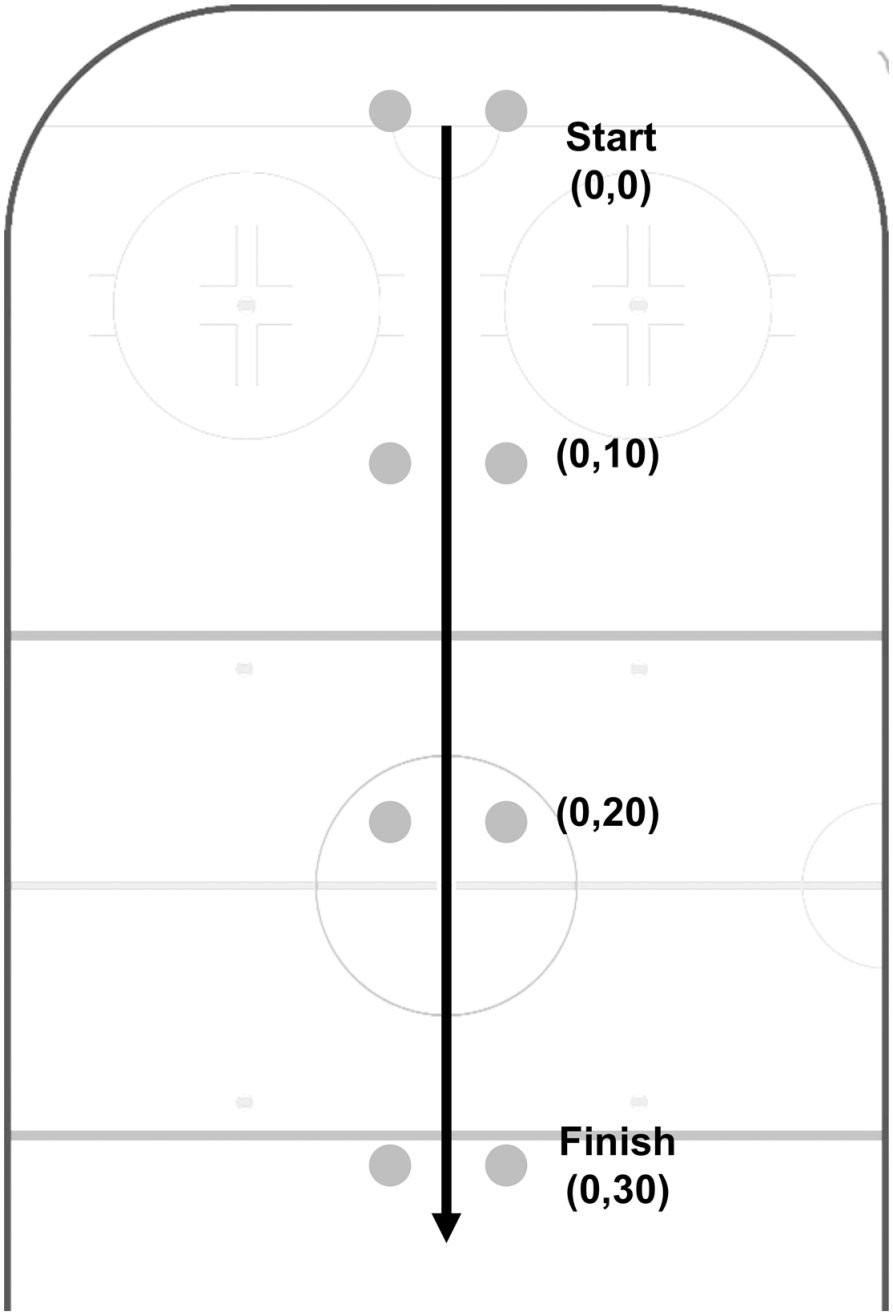
Diagram of the test setup for the 30 m maximum effort forward skating drill. Grey circles represent the position of timing light gates. Co-ordinates are medio-lateral and anterior displacements from the start position (0, 0). The direction of motion is shown by the black arrow.

Nine varsity level (High; age 25.7±3.7 years, mass 86.2±7.8 kg, height 180.0±4.9 cm) and nine recreational hockey players (Low; age 36.9±5.3 years, mass 86.3±13.8 kg, height 177.8±5.6 cm) participated in this study at the Olympic Oval ice hockey rink at the University of Calgary. Groups were similar in height and weight, but there was a significant difference in age between groups (*p*<0.05). The study was granted ethical approval by the University of Calgary’s Conjoint Health Research Ethics Board. All participants were free from injury at the time of participation. They were informed of the risks involved in the experimental protocol and signed an informed consent form in accordance with the University of Calgary’s Conjoint Health Research Ethics Board. All subjects’ skates were sharpened to the same hollow by the same person prior to data collection. They performed 15 repetitions of a 30 m maximum effort forward skating drill (Fig 1).

### Instrumentation and Measurement Systems

A 3D accelerometer (ADXL 78, Analog Devices, USA) with a measuring range of ±35g was placed on the right skate at the center of the chassis. Voltage signals from three orthogonal axes of the accelerometers were sampled at 2400 Hz. The signals were subsequently converted to units of gravity (g) by calibrating the voltage outputs of the accelerometer through a 180° range of motion against a hand held goniometer. The accelerometer was placed on the chassis for stride-detection purposes i.e. quantifying vibrations on the skate when the blade was in contact with the ice.

Muscle activity was measured from the muscle bellies of the right leg for the tibialis anterior (TA), medial gastrocnemius (MG), vastus medialis (VM), vastus lateralis (VL) and gluteus medius (GM) using Ag-AgCl bipolar surface electrodes with a 10 mm diameter and 22 mm inter-electrode spacing (Biovision, Wehrheim, Germany). Electrodes were secured to the skin with Cover-Roll stretch tape (Beiersdorf AG, Hamburg, Germany) after the skin was marked, shaved and cleaned. Voltage signals from EMG electrodes were pre-amplified at the source and sampled at 2400 Hz.

Plantar pressure distribution within the skate boot was measured using the Novel Pedar-X pressure measurement system (Novel Electronics, Minnesota, USA). Instrumented insoles which contained 99 pressure sensors were placed bilaterally into each skate and pressure from each sensor was sampled at 90 Hz. Pressure values were zeroed with the foot inside the skate, laces tied, and skate lifted off the ground.

Angular displacements were measured at the knee in the sagittal plane, and at the hip in the frontal and sagittal planes, using uniaxial and biaxial electrogoniometers (SG150 Twin Axis, Biometrics Ltd, Newport, UK), respectively. Voltage signals from the goniometers were pre-amplified at the source and sampled at 2400 Hz.

Performance times were measured using timing light gates (Brower Timing Systems, Draper, UT, USA) positioned according to Fig 1. This enabled the times for the first 10 m of the sprint (i.e. acceleration phase), final 10 m of the sprint (i.e. steady-state phase), and the full sprint to be recorded.

### System Synchronisation

The accelerometer, EMG electrodes and electro-goniometers were directly connected to a data acquisition (DAQ) unit (Biovision, Wehrheim, Germany). Plantar pressure data was collected independently through custom Novel Pedar-X software (Novel Electronics, Minnesota, USA), and was time synchronized to EMG, acceleration and goniometer data during post-processing. This was done using a synchronisation pulse which the plantar pressure system sent to an open channel in the DAQ unit each time data acquisition was initiated. This pulse caused the signal in the channel to increase its voltage from 0 to >2 mV each time plantar pressure recording was initiated. This information was used to align the plantar pressure data with the raw voltage signals. Pressure data was then up sampled to 2400 Hz through linear interpolation, to match the sampling rate of the DAQ unit.

**Fig 2:**
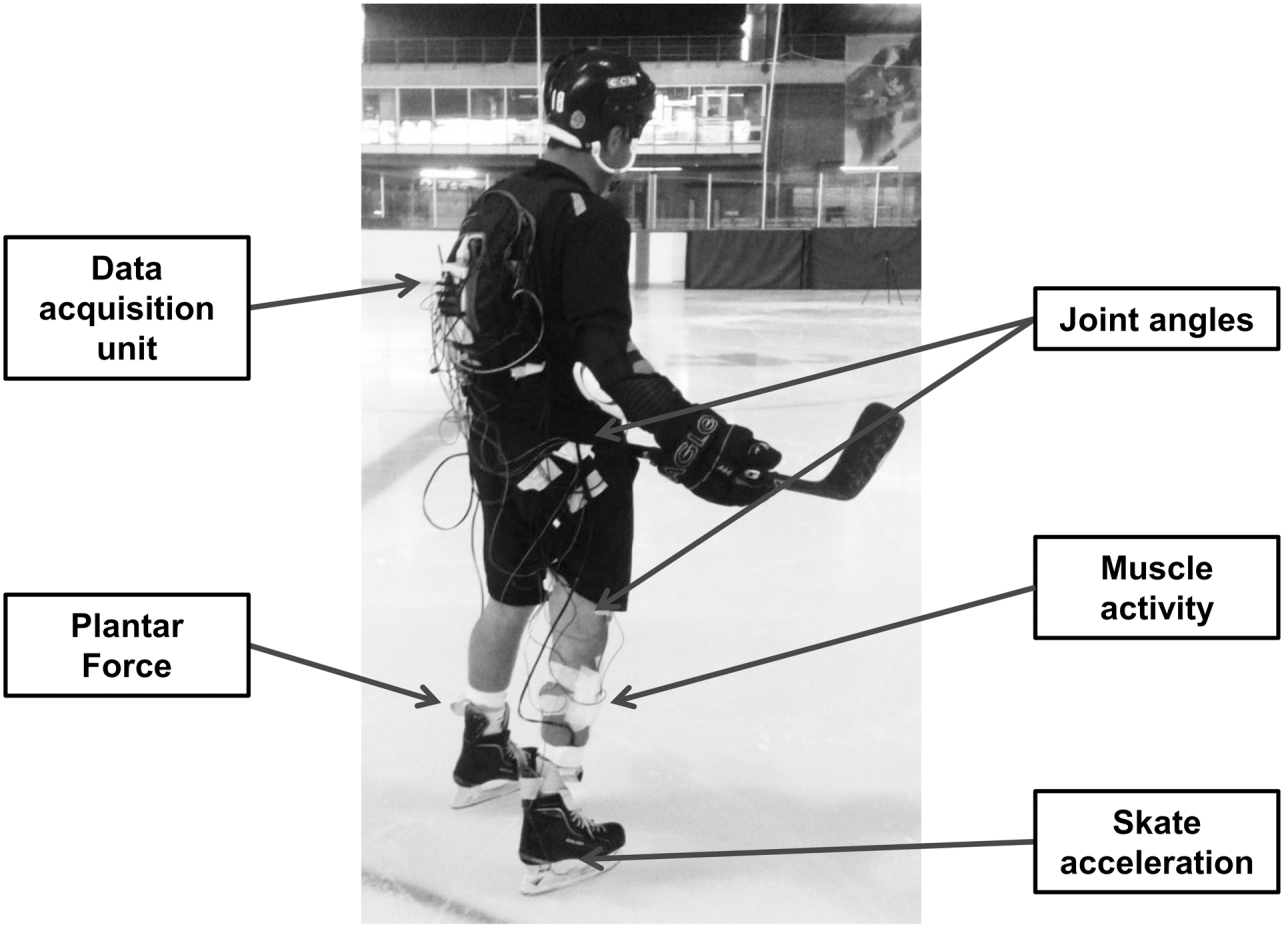
Exemplary set-up of subject following full instrumentation. Subject fitted with EMG electrodes, electrogoniometers, accelerometers, instrumented insoles, and data acquisition system. The participant pictured here gave written informed consent, as outlined in the PLOS consent form, to the publication of their photograph.

Fig 2 shows an exemplary setup of a subject with all sensors and electrodes fixed to the body and connected to a backpack. The individual depicted in the figure provided written consent regarding the publication of their photograph, in compliance with PLOS ONE’s figure policy on depictions of humans. The backpack contained the DAQ unit, analog-to-digital converter, and tablet (Acer Iconia W 510, Acer Inc., Taipei). Data from the analog-to-digital converter was recorded with the tablet and a wireless connection was formed with an external laptop, which controlled data acquisition and recording. The plantar pressure system’s data-logger and battery were also contained in the backpack and data acquisition of this system was initiated through a remote trigger. The total mass of the backpack carried by the hockey player was approximately 4 kg.

### Data Analysis

**Fig 3:**
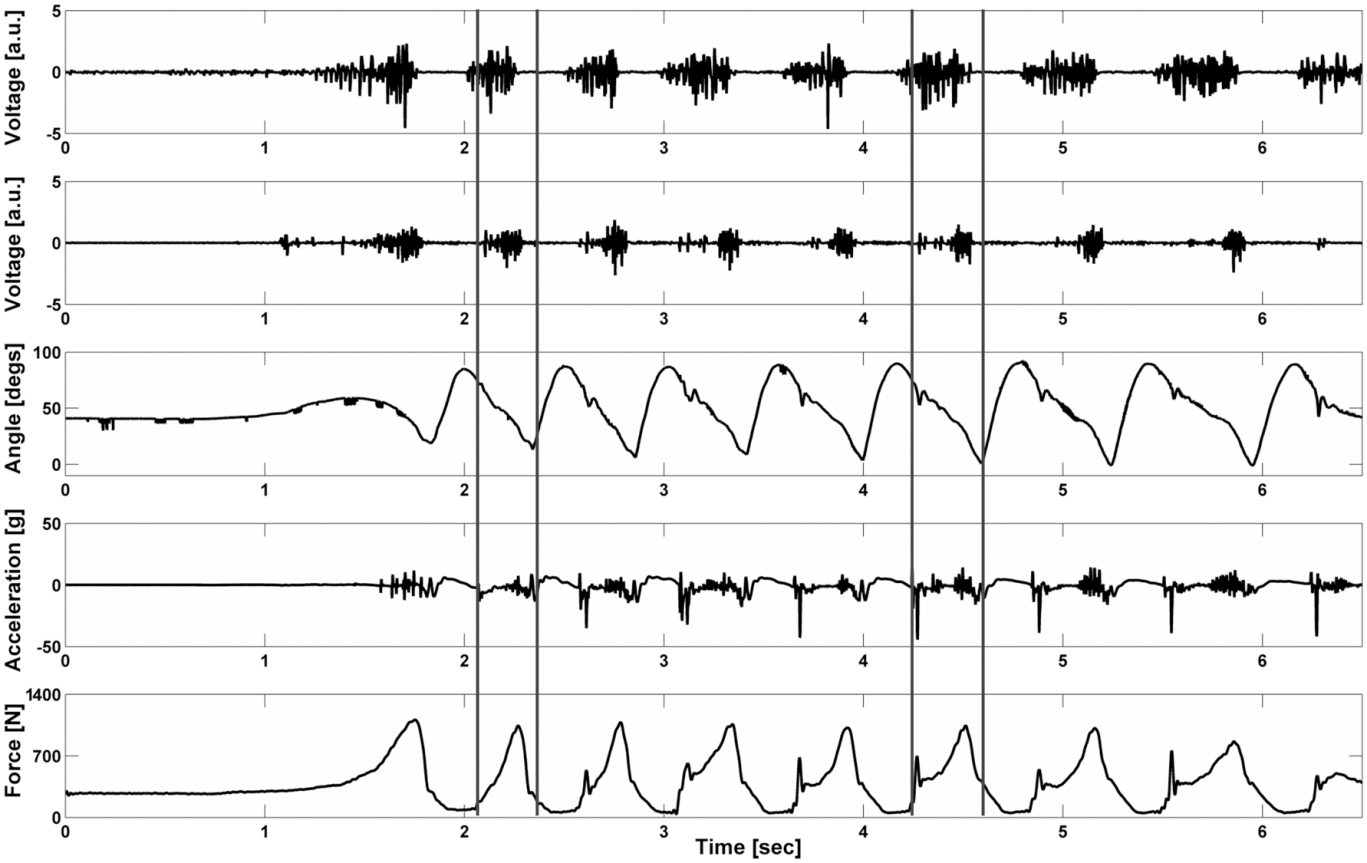
Synchronised data streams taken from one representative forward skating trial of a high level hockey player. Subplots from top to bottom are as follows: EMG of vastus medialis, EMG of medial gastrocnemius, sagittal plane knee angular displacement, vertical acceleration of skate chassis and total plantar force. Data contained between solid vertical lines represent the portions of data analysed i.e. *acceleration* stride and *steady-state* stride.

All biomechanical data from the second and sixth strides of the forward skating drill, where the skate was in contact with the ice, were extracted and time normalised according to the ice contact phase (% ice contact). These strides were selected as they represent the *accelerative* (ACC) and *steady-state* (SS) portions of forward skating [9]. Specifically the ice-contact phase was extracted, as this is where force production and muscle activity are greatest. Ice contact was determined by the sudden onset and offset of high vibration amplitudes in the skate chassis accelerometer signal (Fig 3).

A wavelet analysis technique with 13 non-linearly scaled wavelets (centre frequencies ranging from 6.9 Hz to 542 Hz) was used to resolve the raw EMG signals into time-frequency space [10]. Total EMG intensity was summed across wavelets 2–11 in order to analyze the power of the signal. No maximum voluntary contractions were performed, therefore EMG waveforms of every muscle was normalised to the steady-state stride’s peak intensity measured for that muscle. As such, potential changes in muscle recruitment strategies across strides could be examined across stride types, but inter-group comparisons could not be made.

**Fig 4:**
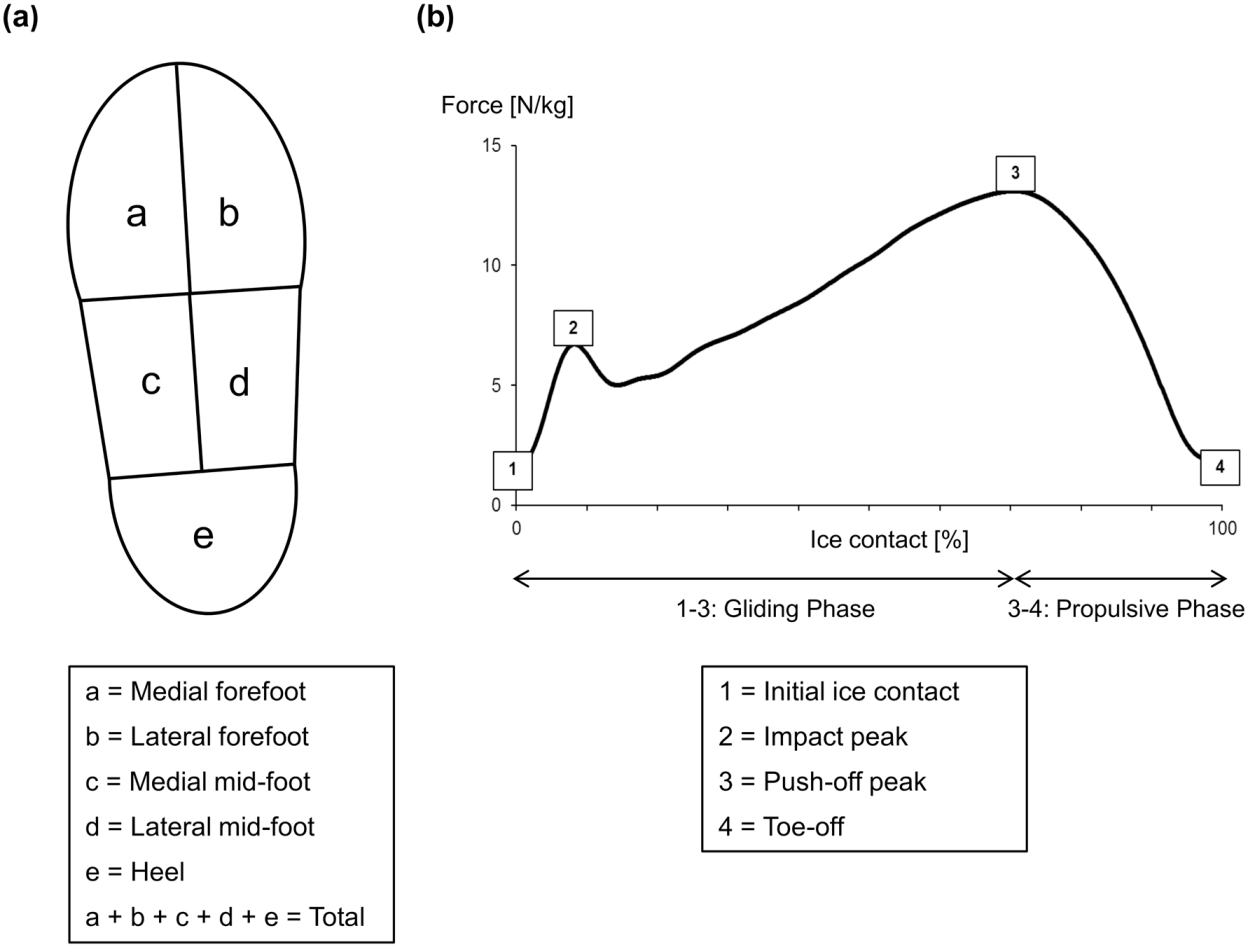
(a) Five masked sections of the right instrumented insole and (b) Total plantar force normalised to the ice contact phase. Force output from five sections of the foot summed across the insole to give total force normalized to body mass. Ice contact phase is spilt into the *gliding phase* and *propulsive phase*. Kinematic data is extracted at points 1, 2, 3 and 4.

Plantar pressure data was analysed by converting each of the insole’s 99 pressure values (Pa) to force (N) based on the area of each cell. Force at each cell was summed to create five distinct segments of the foot. These included medial forefoot, lateral forefoot, medial mid-foot, lateral mid-foot, and heel segments (Fig 4a). At each of these segments, mean force and peak force values were extracted. Additionally, the impact peak and the active peak of the summed plantar force (i.e. summation of five segments) were also analysed. All force values were normalised to body mass so that inter-subject comparisons could be made (Fig 4b).

Electro-goniometer data was filtered using a fourth order low pass Butterworth filter with a cut-off frequency of 20 Hz. Linear calibrations of the electro-goniometers were performed for the sagittal and frontal planes using a handheld goniometer, and was used to convert all raw voltage outputs recorded during dynamic trials into degrees. To account for individual anthropometric differences as well as goniometer placement, a static neutral trial was collected for 10 seconds and then subtracted from each dynamic trial. Six variables were used for the kinematic analysis of the forward skating trials: (i) angle at initial ice contact, (ii) angle at impact peak, (iii) angle at push-off, (iv) angle at toe-off, (v) joint range of motion (ROM) and (vi) maximum angular velocity from push-off to toe-off (i.e. propulsion). Total summed force from the plantar pressure data was used to determine the timing events of these variables (Fig 4b). Based on the total plantar force, initial ice contact to push-off peak was termed the *gliding phase*, and push-off peak to toe-off was termed the *propulsive phase*.

### Statistical Analysis

Coefficient of multiple correlation (CMC) is a statistical measure of waveform similarity that has been used to evaluate repeatability of waveforms in gait analysis [11]. Garofolo et al. [12] interprets CMC reliability as follows: 0.65<CMC<0.75 is moderate, 0.75<CMC<0.85 is good, 0.85<CMC<0.95 is very good, 0.95<CMC<1.0 is excellent. CMC of EMG total intensity waveforms, joint angle waveforms and total plantar force waveforms across 15 ACC and SS strides was calculated in order to evaluate intra-subject repeatability and system reliability.

Statistical comparisons between stride types (ACC vs. SS) and caliber (High vs. Low) were performed in SPSS (version 20, IBM Corporation, New York, USA). Group means and standard deviations of all variables were computed, and normality of the data was tested using the Shapiro-Wilk test. A two-way mixed model ANOVA was run to determine whether differences in biomechanical variables were statistically significant across caliber and strides. Significance level for all tests was set at *p*<0.05.

## Results

### Reliability

Mean and standard deviation CMC values across all variables, strides and caliber are shown in Table 1. The CMC of EMG across all measured muscles varied from 0.67 to 0.88. This represents, on average, ‘moderate’ to ‘very good’ intra-subject agreement across trials [12]. Joint angles and plantar force waveforms showed ‘very good’ to ‘excellent’ agreement across fifteen trials, as demonstrated by CMC values of ≥ 0.91.

**Table 1:** Coefficient of multiple correlation (CMC) values (average with standard deviation in brackets) for five muscles recorded from the right lower limb (vastus medialis, gastrocnemius, gluteus medius, tibialis anterior and vastus lateralis), three joint angles (sagittal plane of right knee, sagittal plane of right hip and frontal plane of right hip), and plantar force from right skate.

		VM	MG	GM	TA	VL	Knee Sagittal	Hip Sagittal	Hip Frontal	Plantar Force
ACC	High	0.72	0.70	0.78	0.70	0.78	0.97	0.97	0.94	0.97
		(0.14)	(0.05)	(0.09)	(0.27)	(0.05)	(0.01)	(0.00)	(0.01)	(0.01)
	Low	0.71	0.77	0.88	0.76	0.71	0.97	0.95	0.97	0.96
		(0.07)	(0.13)	(0.01)	(0.05)	0.05)	(0.03)	(0.03)	(0.01)	(0.03)
SS	High	0.68	0.84	0.81	0.69	0.79	0.98	0.97	0.92	0.93
		(0.10)	(0.05)	(0.09)	(0.08)	(0.02)	(0.00)	(0.03)	(0.08)	(0.01)
	Low	0.75	0.88	0.85	0.67	0.69	0.96	0.97	0.91	0.92
		(0.19)	(0.05)	(0.06)	(0.18)	(0.03)	(0.03)	(0.01)	(0.06)	(0.10)

### Forward Skating Performance

Performance times as recorded by timing light gates are shown in Table 2. High were faster than Low in all aspects of the forwards skating task, completing the ACC phase, SS phase, and consequently the full 30m sprint in significantly quicker times (*p*<0.05). There was a significant interaction effect between *caliber* and *phase*, where Low had a bigger difference between ACC and SS phase times, compared to High (*p*<0.05).

**Table 2:** Performance times of the acceleration phase, steady-state phase, and total sprint distance, of maximum effort 30m forward skating. Mean (SD).

	Caliber	Acceleration	Steady-state	Total Sprint
Time [s]	High	1.93	1.14	4.42
		(0.03)	(0.02)	(0.10)
	Low	2.26	1.23	4.95
		(0.06)	(0.02)	(0.26)

### Muscle Activity

**Fig 5:**
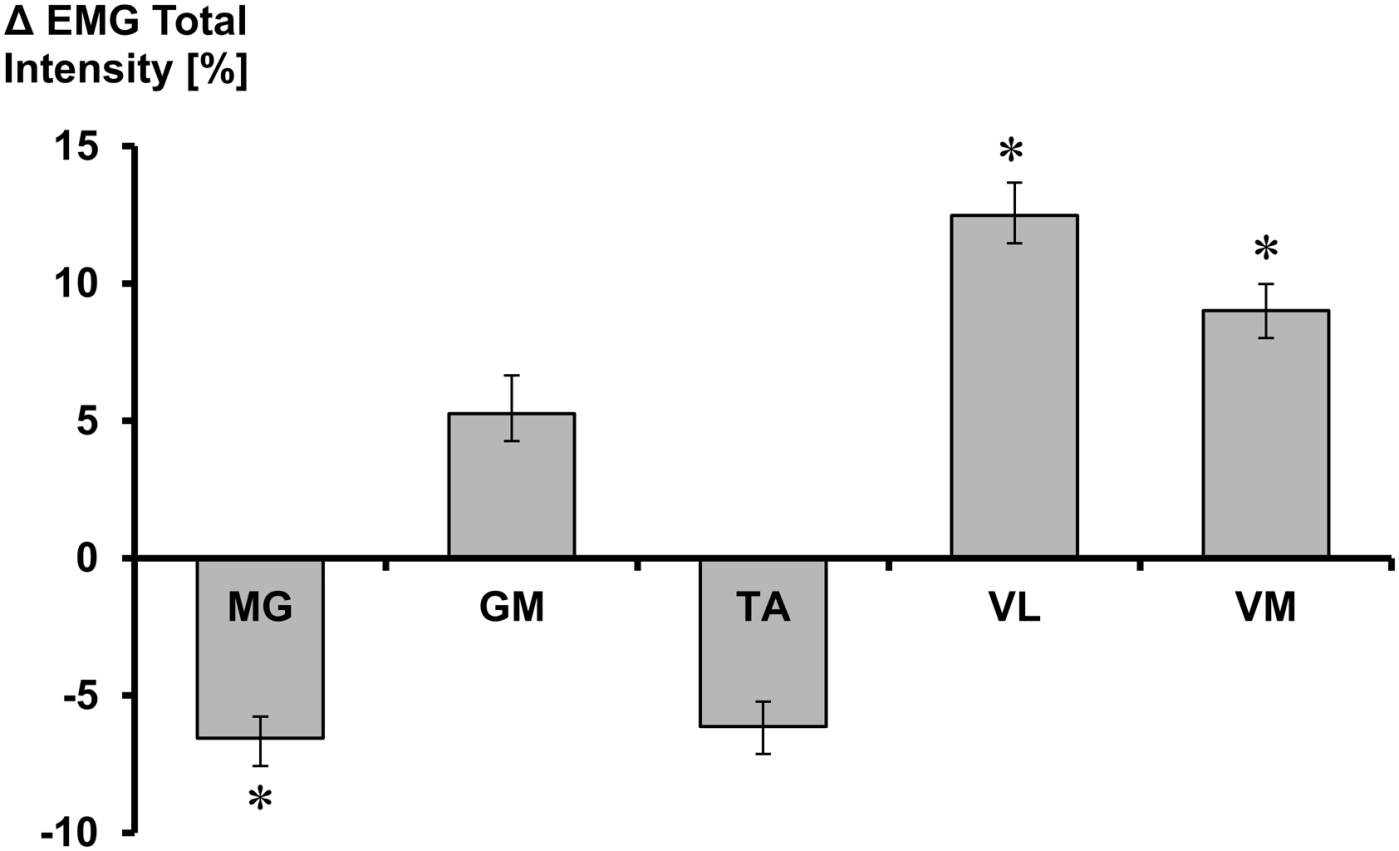
Changes in total intensity of muscle activity between acceleration and steady-state strides. Negative value indicates greater activity during acceleration stride; positive value indicates greater activity during steady-state stride. MG is medial gastrocnemius, GM is gluteus medius, TA is tibialis anterior, VL is vastus lateralis and VM is vastus medialis. Mean and SD. * = statistically significant difference (*p*<0.05).

Percentage difference in total EMG intensity during ice contact, between ACC and SS is shown in Fig 5. A positive value indicates a greater total intensity during SS stride compared to ACC stride. The opposite is true for a negative value. When directly comparing the five muscles between the two strides, MG activity was seen to be significantly greater during ACC compared to SS (*p*<0.01). On the other hand, VL and VM muscles exhibited greater muscle activity during SS (*p*<0.01). There were no significant differences between the SS and ACC for the GM and TA muscles.

### Plantar Force

**Fig 6:**
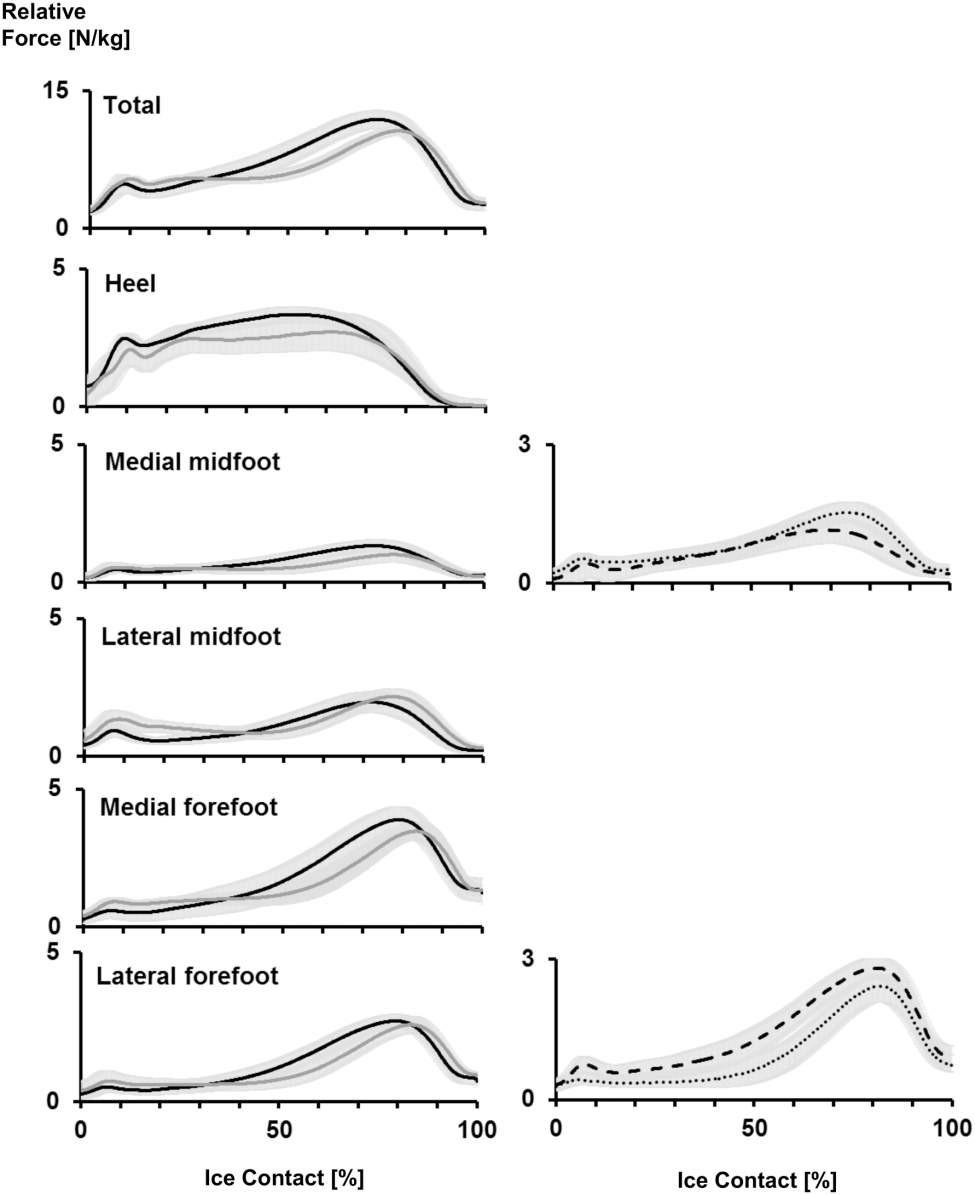
Mean plantar force output waveforms. Left column is mean ± standard deviation across all subjects for total force, heel force, medial mid-foot force, lateral mid-foot force, medial forefoot force, medial forefoot force and lateral forefoot force for acceleration stride (solid black line) and steady-state stride (solid grey line). Right graphs are mean ± standard deviation averaged across both strides for High (thick dashed line) and Low (dotted line).

Fig 6 shows the mean force outputs at each of the five segments of the insole and the total plantar force, depicted over the duration of ice contact for both the ACC and SS strides. During both stride types, High generated larger mean forces at the lateral forefoot than Low (*p*<0.05), while Low generated larger peak forces than High at the medial mid-foot (*p*<0.05).

The push-off peak of the total plantar force curve was significantly larger for ACC stride (*p*<0.01). As such, the mean total force across the insole was also significantly greater during ACC by 0.57 N/kg (*p*<0.01). The lateral forefoot and medial mid-foot segments both exhibited a significantly greater mean force during ACC. However, the lateral mid-foot demonstrated significantly greater mean values during SS stride (*p*<0.05). At the heel, there was a significantly greater mean force during the ACC strides by 0.33 N/kg (*p*<0.01).

### Joint Kinematics

**Fig 7:**
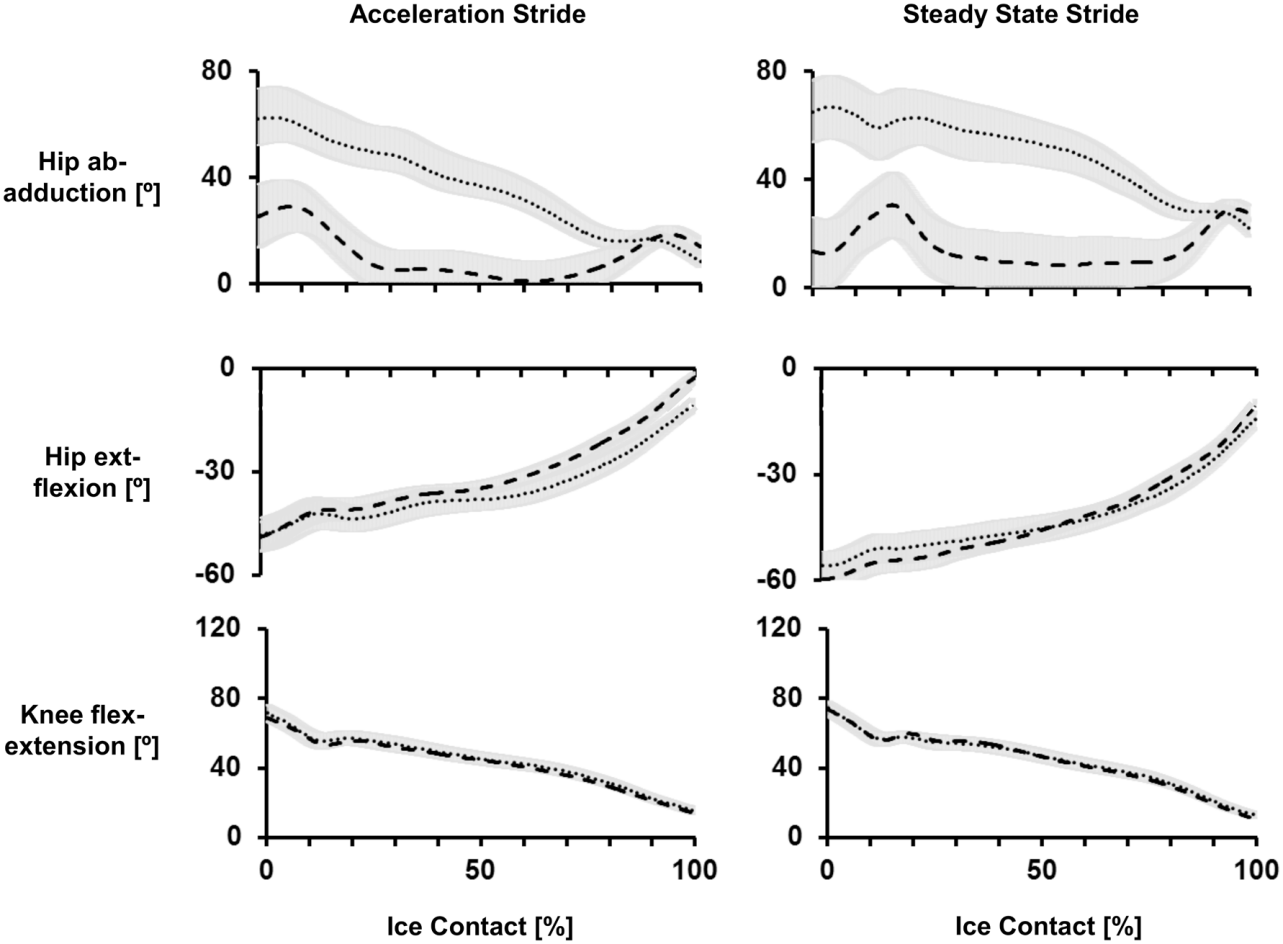
Mean hip frontal, hip sagittal and knee sagittal plane angle waveforms. Left column is mean ± standard deviation of acceleration strides averaged across High (thick dashed line) and Low (dotted line). Right column is mean ± standard deviation of steady state strides averaged across High (thick dashed line) and Low (dotted line). Hip abduction, hip extension and knee flexion were defined as positive.

**Fig 8:**
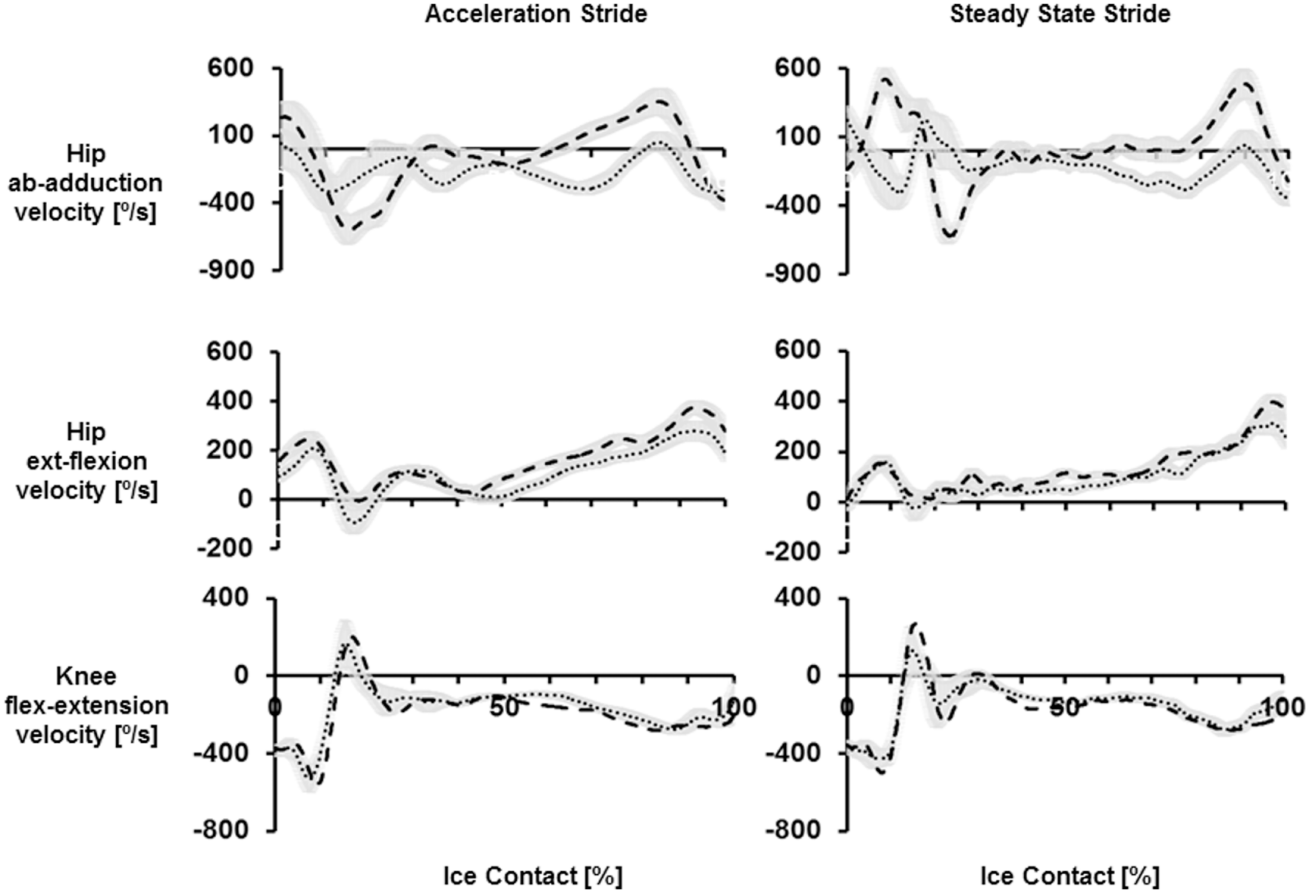
Mean hip frontal, hip sagittal and knee sagittal plane angular velocity waveforms. Left column is mean ± standard deviation of acceleration strides averaged across High (thick dashed line) and Low (dotted line). Right column is mean ± standard deviation of steady state strides averaged across High (thick dashed line) and Low (dotted line). Hip abduction velocity, hip extension velocity and knee flexion velocity were defined as positive.

Angular displacements and angular velocities of the hip (frontal and sagittal) and knee (sagittal) across both strides and calibers can be seen in Figs 7 and 8. During the gliding phase of both strides Low exhibited a more abducted hip compared to High. This was shown by greater hip abduction at initial contact of ACC stride (*p*<0.05), as well as greater hip abduction at initial contact and push-off of SS stride (*p*<0.05). During the propulsive phase High exhibited greater hip abduction velocities across both stride types (*p*<0.05). Overall, a greater hip abduction ROM and hip abduction velocity during the propulsive phase was observed during SS strides compared to ACC.

Regarding hip angles in the sagittal plane, High demonstrated greater hip extension at toe-off across both stride types. This corresponded with their greater hip extension velocity during the propulsive phase (*p*<0.05). During SS strides High also demonstrated a greater hip ROM than Low, where they initiated ice contact with greater hip flexion and ended it with greater hip extension. Overall, ACC strides demonstrated greater hip extension angles throughout the stride, compared to SS.

The only caliber difference seen at the knee was due to High expressing greater knee extension velocity compared to Low during the propulsive phase of ACC (*p*<0.05). With respect to different phases of the sprint, the knee during ACC strides was more extended at ice contact but less extended during push off and toe off, resulting in a smaller range ROM compared to SS.

## Discussion

The goals of this study were, firstly, to quantify the reliability of a mobile data acquisition method for analysing on-ice hockey skating. Subsequent goals were to apply the developed data acquisition method to examine changes in hockey skating biomechanics across progressive phases of maximum effort forward skating, and to identify the biomechanical variables that have the largest contribution to high level skating performance.

The study was successfully performed on ice, which represents the natural environment for ice hockey. Although multiple sensors were fixed to their skates, lower limbs and torso, and a backpack weighing approximately 4 kg was worn, participants gave subjective feedback that they felt no constraints on their skating style. Therefore, the collected data is considered realistic and representative of each individual’s skating technique and performance.

Excellent reliability was observed for measures of joint kinematics and plantar force, while EMG total intensity waveforms demonstrated moderate to very good agreement for each of the five measured muscles. High CMC values, particularly when measuring force and joint kinematics, highlights the ability of the current on-ice measurement approach to record data reliably. Lower reliability of EMG total intensity waveforms were expected, given the higher inherent variability that is associated with EMG measures [13]. Joint kinematics and plantar force application technique remained relatively consistent across trials for a given subject, and this was expected, given that during cyclic activities such as running, muscle activity is usually what is modified in order to maintain a preferred direction of movement i.e. to maintain joint angle trajectories [14]. As such, joint kinematics and plantar force application technique remained relatively consistent across trials for a given subject, whereas EMG showed greater variability across trials. This difference in reliability across measured variables further emphasises the importance of a comprehensive approach of measuring a multitude of variables, when studying hockey skating biomechanics.

Examining the differences in technique between the accelerative and steady state strides provided important information regarding two fundamental phases of ice hockey skating, and how skating technique changes in accordance with increased skating speed. Previous studies of speed skating and ice hockey have shown that as skating velocity increases i.e. players transition from ACC to SS, technique evolves from a running-like motion to a gliding motion, with greater external rotation and abduction of the lower limbs becoming more evident with each stride [2,8]. In support, this study demonstrated a large emphasis on hip extension during ACC strides, and as the subject increased their skating velocity, that emphasis transitioned to hip abduction. This was shown through greater hip extension ROM during ACC strides, and greater hip abduction ROM during SS strides. Therefore, an important movement strategy in ice hockey appears to be the transition from hip extension to hip abduction as skating velocity increases, and this would facilitate the transition to a gliding motion that the hockey player experiences during steady state skating.

In addition to changes at the hip, knee kinematics was also seen to significantly change across strides. In accordance with Lafontaine (2007), knee ROM increased with the number of strides. In other sports, increased lower limb joint ROM have been shown to be key determinants of high performance, particularly through greater magnitudes of joint flexion at the start of a new stride [15]. Therefore, greater knee ROM, achieved primarily through greater knee flexion at initial ice contact, during SS supports the relationship between large joint flexion amplitudes and greater skating velocity. Increased muscle activity exhibited by the VM and VL knee extensor muscles were likely an important mechanism which contributed to greater knee extension during the propulsive portion of the SS stride. Consequently, it might be suggested that high skating velocities can be achieved through appropriate training of the knee extensor muscles, which then allows for greater joint ROM. The lower knee extension angles seen during ACC strides may also be explained by significantly greater medial gastrocnemius activity during those strides. Flexion of the knee is a secondary function of the gastrocnemius, thus significantly greater activity of that muscle during ACC may have contributed to their lower knee extension range. At the ankle joint, the gastrocnemius acts as a powerful plantar-flexor, and this is necessary during ACC to enable subjects to push off from a static position to perform their ‘running-like’ motion. Furthermore, ankle plantar-flexor activity has been found to be a primary contributor to ground reaction forces during the propulsive phase of running [16]. This was reflected in the current study’s in-skate plantar-force data, where push-off forces i.e. peak push-off and mean force, were found to be significantly larger during ACC strides. Therefore, during ACC strides when the subjects push-off from standing, there appears to be a dominant plantar-flexor activity, which may serve to restrict knee ROM and increases plantar force applications. However, during SS strides, increased knee extensor muscle activity provides the mechanism for greater joint extension velocity and therefore, greater skating velocities.

Changes in movement strategies during the forward skating task provided some important biomechanical insight towards fundamental aspects of skating technique. However, isolating the differences between High and Low caliber skating can provide a biomechanical understanding of the variables that may contribute to high level skating performance. In terms of overall performance, there was a clear distinction between High and Low groups. High performed the full 30 m sprint 12% faster than Low, and this was achieved by completing both the ACC and SS phases significantly faster (15% and 7% respectively). It has been shown in a study of speed skating, which also measured EMG, force and kinematics, that elite speed skaters are able to produce a better performance than a lower level group due to their ability to generate larger net joint moments, and thus higher power output [17]. Further speed skating studies have directly related greater performance levels with higher power output and work done during the stride push-off phase [18]. In this study, there was a significant stride-caliber interaction effect, which saw Low with bigger differences between ACC and SS phase times compared to High. As such, the results of this study show that caliber differences are most prominent during ACC, where High were able to take advantage of a faster sprint start, potentially through a more rapid rate of force development resulting in greater power output during the initial accelerative strides.

In addition to differences in performance across groups, the current study showed High to exhibit greater hip adduction angles at initial contact compared to Low, which suggest they bring their leg in towards their midline more rapidly at the initiation of each new skating stride. This enabled them to make use of the greater ROM and thus generate a greater hip abduction velocity during the propulsive phase. The greater hip abduction velocity during the propulsive part of the stride is particularly important during SS where maximum velocity is reached, with greater external rotation and abduction of the lower limbs becoming more evident with every stride (Lafontaine, 2007). Therefore, greater hip abduction velocity during the propulsive phase of a skating stride is indicative of high velocity skating, as exhibited by the High group. Previously, no differences in frontal plane hip kinematics had been identified between high and low caliber hockey players [4]. However, this was investigated on a skating treadmill, where hip abduction, particularly in taller athletes, may be limited by the width of the treadmill belt. As such, the results of this on-ice study of skating biomechanics emphasize the importance of recoding ice hockey movements in the real, unconstrained playing environment.

In addition to frontal plane kinematics, High also demonstrated a greater hip extension at toe-off, which contributed to an overall greater ROM. Greater ROM of the hip and the knee are purported to result in greater extension velocities during the ice-contact phase of a skating stride, and therefore greater force application during propulsion [8]. There was no caliber difference in knee ROM, however, knee extension velocity during the propulsive phase was significantly greater in the High group. The greater rate of knee extension exhibited by High is likely due to a more rapid rate of force development and power production, which contributes to their increased skating speed [4]. Plantar forces measured by instrumented insoles were least effective at identifying caliber differences. Total force across the insole did not differ across player caliber, although this may be because force output was normalized to participants’ body mass. However, some caliber differences were seen in the plantar force application patterns across the insole. During SS strides, forces at the forefoot segments were greater in High caliber players, whereas Low demonstrated larger forces at the mid-foot. This suggests that a possible characteristic of good skating performance is to push-off with the toes during the propulsive portion of the stride, rather than distributing plantar forces across the mid-foot and heel segments. This may be a function of greater hip extension exhibited by the High level group, where the greater the degree of hip extension, the greater the use of the forefoot to allow the player to push-off effectively.

The successful implementation of a reliable on-ice mobile measurement approach offers potential for athlete monitoring, biofeedback and training advice. The results of this study identified the accelerative phase as a key portion of forward skating which most strongly separates the performance of High and Low level hockey skaters. Furthermore, biomechanical variables which best discriminate High and Low level hockey skating performance were identified. While it cannot be inferred that these variables directly influence skating performance, they certainly pave the way for future study designs which specifically address the development of hockey skating coaching tools.

## Conclusion

The implementation of a mobile measurement approach allowed for a successful on-ice data collection of lower limb biomechanics during forward ice hockey skating. Good to excellent reliability across trials was demonstrated, suggesting the method is sufficiently sensitive to examine hockey players on an individual basis. The study’s results provided fundamental knowledge of ice hockey biomechanics with respect to skating performance, showing biomechanical differences across successive strides. Biomechanical differences were also observed across player caliber, thus providing an understanding of specific variables that best discriminate high and low level hockey skating. Previous investigations of skating biomechanics have been constrained to investigations of only forward skating due to restrictions imposed by their methodologies. While the current approach also only investigated forward skating, the method employed is mobile and wireless, and does not impose any movement restrictions. Therefore, further steps would be to apply this methodology to tasks such as backwards skating, crossovers, two-footed turns and hockey stops. An important future application of this system would be for athlete monitoring purposes, to provide coaches and athletes with real-time biofeedback and subsequent training advice.

## Supporting Information

S1 File Supplementary Data. EMG, plantar pressure and kinematic data across all individual high level and low level ice hockey players.
(XLSX)
